# Innovative Housing Policy and (Vulnerable) Residents’ Quality of Life

**DOI:** 10.3389/fpsyg.2022.751208

**Published:** 2022-02-11

**Authors:** Joanna Frątczak-Müller

**Affiliations:** Faculty of Social Sciences, Institute of Sociology, University of Zielona Góra, Zielona Góra, Poland

**Keywords:** positive public policy, multi-level government, social housing, vulnerable people, quality of life

## Abstract

The subject of the current study is the process of implementing the social housing policy and its impact on increasing the quality of life (QOL) of vulnerable people. The analysis is related to the Social Housing Program introduced by the city of Gdańsk (Poland). The study has been carried out using the case study method with the use of document analysis, analysis of existing data, and five in-depth interviews with program managers. The theoretical framework has been developed around three major explicatory perspectives: multi-level governance (MLG), QOL, and social exclusion. Meeting the housing needs is one of the primary tasks of local governments. If such a policy is to be called positive, it should be combined with social policy tasks related to social inclusion, and it should be carried out through partnerships forming housing cooperation networks. A characteristic feature of the cooperation is the cross-sectoral nature of the actors. The results have revealed a significant positive relationship between the use of social housing tools and the quality of satisfying the living needs of the residents. This study contributes to the discussion by revealing the role of social work and social and professional activation in meeting housing needs. The results suggest that a combination of housing and social policy measures can help vulnerable people develop social competencies conducive to housing maintenance and increase the social cohesion of local environments.

## Introduction

Living space is a basic condition for running a household and a family life. By satisfying basic human needs, it fulfills many other functions simultaneously: improving quality of life (QOL), increasing motivation for social activities, supporting intellectual development, minimizing frustration, and building a sense of security. It is one of the fundamental factors in making decisions about starting a family (Mulder, 2006; Cunningham et al., 2019). Research shows that having easy access to housing results in beneficial social phenomena, reducing poverty and exclusion circles, opening prospects for free migration, and reducing disproportions in social stratification (DFID, 2005; Baptista and Marlier, 2019). It creates social bonds of people living in the neighborhood and generates the social activity to develop the surrounding space (Abbé Pierre Foundation and FEANTSA, 2019).

However, housing is costly, especially for low-income people. In this situation, the exclusion does not only include physical problems with the apartment (the roof over the head) but also issues with establishing social relationships and problems with administrative and legal regulations (GSHP, 2016). This view implies that limited access to housing degrades the meaning of an individual’s entire life, negatively influencing their emotional, psychological, and social behavior. It can also lead to one being placed in care institutions, e.g., 24-h nursing homes, single-mother homes, homeless shelters, and other government-run facilities to support and provide housing for the dependent or needy. Such places take away independence, self-steering (Vrugt and Koenis, 2002), privacy, and force residents to live in a unified, formal environment (Goffman, 1961). In such cases, the topics of confinement are also discussed. The term “confinement” has been studied in the anthropological literature in contexts relating to hospitals, retirement homes, prisons, and detention centers (e.g., Chapman et al., 2006; Ramkissoon, 2020a). It is also suggested that place confinement can also be conceptualized as a perceived threat to social mobility (Ramkissoon, 2020a).

Even if existing methods of managing housing resources in communities were supposed to counteract the above situation, they did not bring about positive changes. People at risk of social exclusion and with lower social competencies could not maintain their apartments and care for their condition (Bretherton, 2017; Council of Ministers, 2019). The location of these flats in disadvantaged districts also sustained the creation of poverty circles, social passivity, and the acquisition of inappropriate life patterns by young people (Halpenny et al., 2002). In the present study, we are focusing on examining whether the combination of housing policy with psychological and social work and constant social support for residents protects them from loss of housing and increases the social commitment of their residents. Moreover, some theoreticians have argued that policymaking can positively affect building social cohesion when conducted with the participation of the intersectoral nature of the actors. Considering the concept of multi-level governance (MLG; Piattoni, 2010), we are also examining the impact of introducing social organizations into delivering social services in this housing policy model.

## Methods

### Social Housing Policy

As far as setting priorities in terms of the objectives of social housing policy is concerned, the approach of the European Federation of National Organizations Working for Homeless People FEANTSA (FEANTSA, 2007) should be mentioned. This organization signals that the social housing policy is a way of implementing tasks related to creating conditions for the purchase or rental of a home by all citizens, providing housing resources to people in need, and maintaining appropriate living space. It is a task of public services performed by public institutions, usually of a municipal nature. The main task of the social housing policy is to prevent homelessness and housing exclusion, the so-called lack of a house being defined as rooflessness, houselessness, living in insecure housing, and living in inadequate housing.

Meanwhile, a permanent principle of an ineffective social housing policy is to place people in need in care institutions and other institutional forms of housing that strongly reduces residents’ QOL. Research on homelessness and social exclusion show the following relationship—the longer people remain homeless, the more complex is their process of reintegration into society. According to experts, people exposed to a lack of home receive too little state aid, often limited to the allocation of temporary shelter, which becomes the final solution after some time and causes social exclusion (Abbé Pierre Foundation and FEANTSA, 2019). The principles of allocating social housing resources according to the income criterion do not always reflect “urgent” housing needs (GSHP, 2016; OECD, 2020). The following features characterize the social housing sector: financial support at least at one stage of the life cycle of the housing resources, applicable access criteria for potential users, strictly defined rules of operation (legal regulations) introduced to rationalize activities, transparency of decisions, funds management, and environmental and social standards. The social housing sector presented in this way can function as the ownership or rental sector (Kemeny, 1995; Scanlon et al., 2015; Tang et al., 2017).

### Vulnerable People

The implementation of the above objectives of state policy requires specific instruments regarding people with low economic status or special social groups described as vulnerable or in need (Numans et al., 2021). These include disabled people, long-term unemployed, lonely elderly people, large families, single parents, immigrants and refugees, ethnic minorities, people in danger of eviction, people living in dangerous neighborhoods, as well as young couples and students. All the groups mentioned above experience difficulties in accessing resources leading to their social exclusion or the threat of it (Fernandez-Gavira et al., 2017). It should be noticed that the criteria for identifying vulnerable groups vary significantly in individual countries of the European Union and additionally change over time.

### Multi-Level Governance in Creating Public Policies

Multi-level governance is concerned with decision-making procedures and the regulation of political negotiation processes. Its characteristic feature is the extension of the typical hierarchical course of the decision-making process with nonhierarchical elements. To achieve this, public institutions involve entities representing all sectors: public, non-governmental, and private (Klijn and Skelcher, 2007; Piattoni, 2010; Frątczak-Müller and Mielczarek-Żejmo, 2019) in the decision-making process. This leads to decentralization of management, in which organizations and external experts become important actors. Decentralization activates participants in this process to expand their competencies in creating and implementing public policies (Knoke, 1990; Tausz, 2002). In the social housing policy, in addition to public institutions and their specific departments (social affairs department and housing affairs department), activities of family support canters and social organizations providing social support services are included. A new type of cooperation also refers to the application of the knowledge of external experts. Psychologists, cultural program coordinators, educators, and street workers have an important role to play. The diversity of participants in this cooperation increases the innovation of activities and the inflow of new information (Folke et al., 2003; Granados and Knoke, 2005).

Several studies have provided empirical evidence for the relationship between trust and multi-level collaboration. Institutional trust allows for the implementation of common goals (Ramkissoon, 2020b) because it defines trust as the belief that public institutions are open to dialog and cooperation and will not perform tasks contrary to social expectations. This affects the attitudes of local actors toward local government authorities. Institutional trust is, therefore, an essential condition for intersectoral cooperation. It is also an important feature of civil society (Lovell, 2001). In addition, the standards adopted in it governing participation, the quality of impact and methods of managing cooperation confirm the presence of a relatively high level of procedural justice. It is one of the most critical factors of the willingness to cooperate with a diverse composition of entities (including public institutions; Lind and Tyler, 1997).

### Quality of Life

Quality of life is the degree to which an individual is healthy, comfortable, able to participate in or benefit from life events. It is an integrated system of motivational and aspirational factors, the basis of which are the needs and values of an individual (Nussbaum and Sen, 1993; Costanza et al., 2007; Fedriana et al., 2018). The standard of living depends on individual and social expectations, the type of activity of the individual, and one’s life choices (Danna and Griffin, 1999). Particularly, QOL indicates two possibilities of measurement: (1) objective criterion, defining the relation of the individual’s needs to the resources of the environment and (2) subjective criterion, state of mind of an individual in the process of satisfying needs, resulting from the cognitive assessment of the way of satisfying needs and the related achievements and chances of achieving life goals (Sigry, 2002). QOL has a wide range of contexts related to public policies. For example, it applies to healthcare, housing, and employment policies (Max-Neef, 1992; Buunk and Gibbons, 1997; Massam, 2002).

Concerning having housing resources, an important determinant of well-being is developing a sense of place affect. Place effect has been defined as the affective bond that connects individuals with the environment and promotes psychological restorativeness (Ramkissoon, 2021). Some evidence in the literature shows that an added benefit is also using the site closure of the COVID-19 pandemic as a way to foster social bonding that can then contribute to well-being (Lawton et al., 2009; Williams and McIntyre, 2012). People in confinement settings can start to develop a stronger sense of self-identity with their apartment as the only available space and find that it can fulfill various new functionalities. It is also important to realize its difference from other places. The affect of place will then be associated with a reduced level of stress, promoting well-being (Williams and McIntyre, 2012), contributing to the improvement of the inhabitants’ quality of life (Ramkissoon, 2021).

### Purpose of the Study

The present study performs an in-depth analysis of the implementation of the Gdańsk Social Housing Program for 2016–2023 (GSHP, Program). The Program has been running since 2016. Over 100 people representing about 40 organizations participated in its development. The Program leader and manager of the developed housing policy were the Social Development Department of the Gdańsk City Council. The basic issues discussed in the article refer to three areas: program structure, principles of its implementation, and its effects, with particular emphasis on changes in the quality of life of the participants. It is also important to assess the impact of the applied MLG on shaping public policy. At this point, the thesis is that the GSHP is a stable set of interdependent entities representing the public, private, and social sectors, which implements the principles of the deinstitutionalization process and MLG with the application of stable cooperation patterns, concentrated around needs and social problems within the implementation of public policy. This results in permanent social changes and an increase in the quality of life of the inhabitants.

### Sourcing Foundation and Data Analysis

The present analysis is based on GSHP research with the application of three methods: document analysis, analysis of existing data, and unstructured interviews. The rationale for choosing such a source was: (1) the availability of information about the Program and projects implemented within this framework on websites and (2) the possibility of accessing statistical data obtained by the city council and the organizations cooperating with it on the relevance of the solutions used in the Program and meeting the needs of residents. The analysis of the documents included: (1) legal acts and strategic documents related to GSHP implementation,^1^ (2) financial and organizational reports on the Program operation,^2^ (3) reports on projects realized within the Program framework (e.g., social housing estate Dolne Młyny^3^), and (4) data on the performance of Program indicators.^4^ One of the analyzed documents was also a report on the study of residents’ quality of life (beneficiaries of foster care) in care facilities carried out by the Gdańsk Social Innovation Foundation—one of the Program’s contractors. It was also the organization represented in unstructured interviews.

The construction of the Program defined the direction for the search for participants in unstructured interviews. In total, five interviews were conducted with persons representing the four basic institutions of the Program: (1) Social Development Department, (2) Municipal Services Department of the Gdańsk City Council, (3) Municipal Family Support Centre in Gdańsk, and (4) representative of social organizations—Gdańsk Social Innovation Foundation. We conducted the fifth interview with a representative of the Program management group. The respondents were aged 35–50 (three men and two women). The selection criterion was the knowledge of the Program operation and experience in implementing its tasks. The interviewees worked on the development and implementation of the Program and were identified by the organizations surveyed as people with the deepest knowledge of it. The collected information concerned mainly the principles of the Program implementation, partnership cooperation, decision-making methods, as well as ways of working with program participants and obtaining feedback on the residents’ satisfaction with the results.

Based on the interviews, qualitative data analysis was performed. The analytical process of the collected data from the interviews included three stages: data reduction, data display, and the derivation/confirmation of conclusions. In the transcripts of the interviews, we searched for meaningful concepts and key events. We also performed a repeated reconstruction of the text until the connections between the topics selected for analysis were revealed (Patton, 1990; Silverman, 2016). Audio recordings and transcripts of interviews are stored at the University of Zielona Góra. In the current text, interviews that contain premises for concluding are marked with the letter ‘I’ and a number (e.g., I1).

## Results

### The Origins of the Program

**Figure 1:**
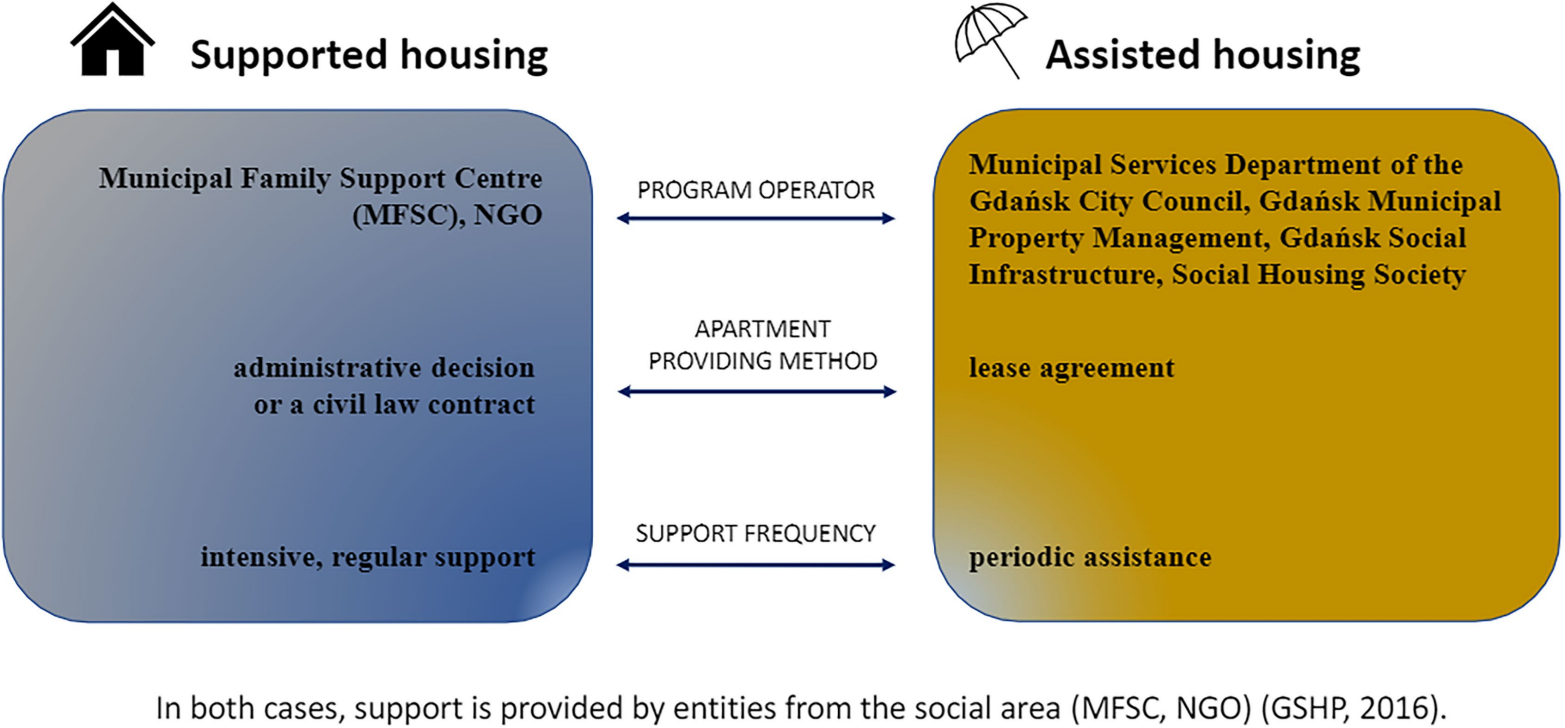
Two models of the Gdańsk Social Housing Program.

The GSHP combines solutions in housing policy with solutions in integration and social assistance, and due to this collaboration, two models of housing have been developed in the local municipal law: supported housing and assisted housing schemes. Although the former has a well-established position in Polish legislation, the latter is a novelty. Its task is to increase the effectiveness of the management of housing resources in the commune and to improve the quality of life of its participants (I2; I4; and I5). The main principles of the program operation are presented in Figure 1. The development of the Program was a response to the housing crisis in Gdańsk. Before the launch of this project (2015), the commune had 16 flats adapted to the needs of seniors, 37 for people with disabilities, six for foster care, 81 for the homeless and excluded from housing, six apartments for addicts and people experiencing violence, and one for immigrants. At the same time, 2,500 families were waiting for council housing and 1,900 families for social housing (GSHP, 2016). A similar situation occurs throughout the country. According to the report of the Supreme Audit Office of 2020, the housing needs of low-income households remained unsatisfied in more than 73% of municipalities. The communes showed an inappropriate rent policy, including non-collection of timely payments. As a result, there was a shortage of funds to cover the operating costs of flats as well as the modernization and renovation (NIK, 2020).

This Program primarily targets people and families threatened with difficulties. The solutions used in GSHP combine housing assistance with the support of a psychologist, lawyer, social worker, or educator in solving everyday life problems. The mechanism of functioning of the Program is as follows: municipal housing services secure the number and quality of housing planned in the Program for people or families in need, and municipal social services provide support to those people or families to reintegrate into society and regain independence in life. This systemic cooperation in housing policy and social policy is also intended to ensure better performance of residents in their responsibilities to care for municipal buildings and to pay rent. “Previously, there was not enough cooperation in council housing, helping to leave the support system after training. And if we managed to get the housing that these people needed, we often had such a problem that these people owed them. Hence, we need to come up with a solution that will be a transition stage between a supported house and a typical council flat. We came up with a program that we called ‘houses with assistance” (I2).

However, the most crucial task of the Program is to create a chance for many vulnerable people and families to function in an intimate environment rather than in the environment of the appropriate institutions. In this way, the principle of deinstitutionalization of social services is fulfilled. Due to this solution, according to one of the interviewees, “finding an assisted apartment almost overnight in a challenging situation is possible” (I2).

### Housing Stock

**Figure 2:**
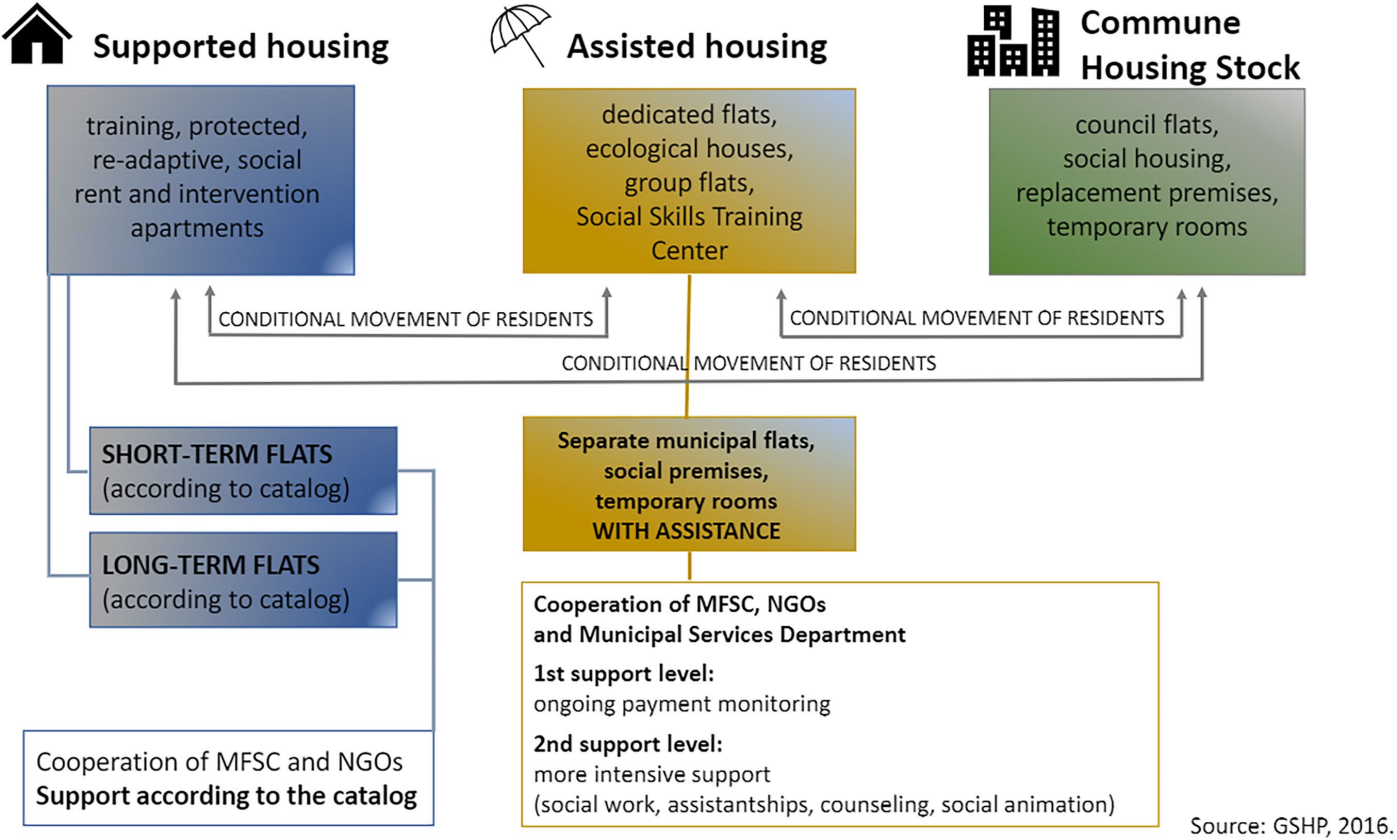
Implementation of supported housing and assisted housing schemes.

The Program is structured in three stages, with the participant being able to move from supported housing, through housing with assistance, and council housing accessible to all residents who meet the income criterion. The connections between these stages are shown in Figure 2. Entering the last stage is seen as the resident becoming independent. Nevertheless, it is not necessary to go through all levels of GSHP. Some residents enter the Program immediately after living in housing with assistance when they face, for example, difficulties in managing the home economy that leads to debts or have experienced violence, which results in the need for psychological support and the development of social competencies. They usually spend about 2 years in the Program. In the case of young people who age out of foster care, independence comes even after a year (I1; I2; and I4). It is also possible to leave the Program and return to it again due to subsequent crises after becoming independent. “It is understandable that the 20-year-old does not want to meet with a foundation employee and that after leaving the orphanage, she/he may want to reject help first. And that is ok; it is about freedom. So she/he has the option not to fulfill the contract, but she/he has to move out. We are trying not to treat it as a failure. Many of such people come back to us. Sometimes after several months, sometimes after several years” (I1). It is also worth pointing out that one can be removed from the Program because of not following its rules and with the possibility of returning to it again after completing social skills training. There are also program participants who will never leave it—people with disabilities or seniors—as it is difficult to expect such an improvement in health or competencies that let them function in the open housing market (I1; I2; I4; and I5).

### Program Recipients

The Program’s activities are aimed at adults who cannot overcome their housing problems with their available resources and possibilities. They are vulnerable people, such as young people growing up in difficult conditions without the support of their parents, people with special needs and disabilities, people at risk of eviction or experiencing homelessness or recovering from addictions, but also victims of domestic violence or random events or traumatic events. Among vulnerable people, there are also people with low professional qualifications, the elderly and lonely, immigrants and national minorities.

### Main Principles of Program Implementation

The implementation of GSHP is based on the principles of cooperation, human dignity, flexibility in the actions taken, trust, and continuous education.

#### Partnership in the Program

During both the stages of the development of the GSHP and its implementation, the emphasis is placed on cross-sectoral cooperation and 95% of the tasks in the Program have been assigned to social organizations, which are responsible for implementing social work, social and professional management, psychological support, or additional social assistance. NGO trainers are also lead trainers who contact program participants, assess their needs, and perform their tasks. It should be noted that not all jobs are commissioned by the community, as some of them appear as the own initiative of social organizations (I2). Similar cooperation affects the recruitment of participants to the Program. “We have very partnership cooperation here. If in our opinion one of our proteges is suitable for living in one of the Municipal Family Support Centre apartments, they do not say no, and if they have another person for this place, we discuss which option will be better” (I1).

#### Individualization of Support

The support provided under the Program is individualized, which is ensured by the diversity of organizations that cooperate with the city and their specialization. Currently (2021), 20 organizations deal with a wide range of social problems. The techniques used to work with residents are also important. The basic rule is to diagnose the participants when they enter the Program. The diagnosis is based on the individual’s goals and not on the shortcomings. It is a concept that derives from idea of nonviolent understanding of Rosenberg (2015).

The second important rule is to provide residents with as many hours of support as they need. If the person studies or works and does not require constant approval, meetings are held once a month. If she/he has more significant needs, the trainer will meet her/him more often. According to the representative of the foundation who participated in the research, this support should never be provided in an apartment. “In typical (highly functioning) families, the psychologist does not visit their home” (I1). The type of support is also adjusted to the needs of the residents. This is the only way to increase the sense of security and quality of life. The analysis done by the foundation reveals that residents who cooperate with it describe maintaining independence and safety as their basic needs, and meeting them requires different methods. There is always the issue of effective budget management and the principle that the rent is to be paid regularly.

In its work, the foundation has adopted a model of the person-centered approach. “We are working on very different things. Some people need to be safe, and they need a lower rent, and that is okay for us. Some people do not fully know what opportunities and prospects are good for them, and we talk about it with their consent. This is one piece. However, some people are in a deep crisis. They come out of violence, from diseases (physical and mental), and here, the work is more intense. We then cooperate with the Municipal Family Support Centre and we make sure that this person has a family assistant, a social worker” (I1). Due to the diversity of organizations working in the Program, this is not the only rule.

### Program Effects

The implementation of the Program started in 2016. At that time, the city’s resources included 147 places (flats/rooms) in various forms of support (supported, training, etc.; I3). At the end of 2020, GSHP had 70 supported places operating under 14 projects with 254 people benefitting from this part of the Program. There were 105 apartments with assistance in five projects, including 48 temporary accommodations at the Social Skills Training Centre. In total, in 2020, 143 people used assisted housing scheme. The analyses conducted by the Gdańsk Social Innovation Foundation show a clear improvement in the social competencies of its proteges and an increase in the quality of life. Since 2016, 55 people have benefited from its help, 28 foster children, 13 partners/spouses, and 14 children. Around 15 people have already left assisted housing, seven of them having obtained apartments from community resources. Five people have rented apartments on the open market. However, three people have chosen their private path and do not want to stay in touch with the foundation (I1).

The foundation’s data show that 100% of people starting work in social enterprises and using assisted rental left the Program and are doing well on the open housing and labor market (I1). This is a significant finding indicating the development of good practice and it indicates the increase of the effectiveness of housing support when combined with supported employment. Currently (2021), 23 out of 28 residents are long-term workers with the rest studying, caring for children, or having limited employment possibilities due to health difficulties. Among all people working long-term (under the foundation’s care since 2016), seven were promoted to a higher position, seven achieved stabilized employment (transition from contract to full-time work), three people started their own company. Of all these people, 10 participated in work in social companies run by the foundation (I1).

Another important success of the Program is the security of residents in the COVID-19 pandemic. As a result of the complicated situation in the local labor markets, some residents began to fall behind on rents. The Program’s employees provided such families with additional support, spread their repayments into installments, or canceled the debts. Furthermore, it turned out that the feeling of loneliness, common in the pandemic, was not noticed in the groups sharing apartments (three-person apartments for young people participating in the Program).

The pandemic, however, made it very difficult to implement one of the younger projects at GSHP—the Housing First project. Its goal is to support the homeless. Its principle is to provide an apartment first and then to implement social work. According to one interviewee, the confinement of people experiencing homelessness in their apartments due to lockdown was like imprisonment, which resulted in difficulties in maintaining housing stability and returning these people to life on the street (I2). The Housing First project led to the additional discovery. Thanks to its implementation, it was noticed that some people in the crisis of homelessness, staying in nonresidential places, are people with intellectual disabilities. It was a surprise both for the Program implementers and the organization supporting the city in terms of the theme of the Housing First project: Entrada—AEIPS – Associação para o Estudo e Integração Psicossocial (I2). The low effectiveness of the support system for people with disabilities in Poland poses an additional challenge in working with these people in this Program.

## Discussion

The subject of the study was the implementation of a social housing policy by the city of Gdańsk. Using the case study method, we are analyzed its structure and performance principles, looking for its strengths, weaknesses, and good practices.

The findings revealed that both program development and management are cross-sectoral. Several institutions and several dozen organizations are involved in implementing the Program and fulfilling their tasks, they draw on their knowledge and experience at the same time applying the principles of their own organizational cultures (McPherson et al., 2001). This meets the principles of decentralization indicated in the literature (Knoke, 1990; Tausz, 2002), differentiating social services (Pestoff, 2012) and reaching service recipients in various places (Fleming and Osborne, 2019). In addition, the multiplicity and diversity of organizations implementing the Program confirm the individualization of support, which is considered key in many studies in the field of social policy and vulnerable groups (Fernandez-Gavira et al., 2017). Such a situation makes the Program flexible but leads to a lack of standardization of the work of the lead trainers. Each of them, using his own experience, develops his techniques and work methods. Currently, the Program has started reviewing these techniques—organizing evaluation meetings for the social organizations. Some of them mention the possible need for a minimum standardization of the working methods used and such an expectation is in line with the institutionalization of associations and will most likely lead to it. This lack of minimal standardization of the organization’s work is also considered one of the limitations to the GSHP implementation.

The results also showed that the Program team is creating a self-learning system that is about establishing patterns of behavior, replicating them, and adjusting them to the needs of the inhabitants. The knowledge generated by its participants is transferred as part of cooperation and a diverse team allows creating new interpretations of the information obtained, which leads to the development of the applied methods of operation and the increase of the adequacy of support (Folke et al., 2003; Granados and Knoke, 2005). This finding is in line with Piattoni (2010) and confirms the use of MLG in the implementation of the Program.

Organizations cooperating in the Program show a fundamental similarity (NGOs with a flat social structure, democratic decision-making, and uncomplicated communication channels), facilitating cooperation. Furthermore, research on MLG emphasized the similarity of organizations connected by this type of cooperation. The feature that brings organizations closer together is, primarily, their type, which is closely related to the legal and organizational framework that determines the ways and scope of their activities (McPherson et al., 2001). Their diversified specializations increase the effectiveness of the Program. Literature proves that an essential element of this cooperation is trust in local institutions (Grzeskowiak et al., 2003; Nunkoo and Ramkissoon, 2011). Trust influences the community’s attitudes toward the policy of local actors and the tendency to carry out common tasks (OECD, 2001). Institutional trust is the basic condition for the implementation of the idea of civic society and local development in such programs as the one presented. The conducted analyzes additionally confirm the findings of the procedural justice concept in the cooperation of social organizations with the local government. According to this approach, actors do not only care about the quality of the final results of their contact with representatives of public institutions. They are also often interested in whether the procedures leading to achieving their goals (consistent with the local community and individual) can be considered fair (Lind and Tyler, 1997). The in-depth interviews showed a high level of conviction about maintaining procedural fairness in actions concerning the program operator and social organizations performing the tasks.

Finally, our findings display the program’s effectiveness in increasing the quality of life of its participants. Data on objective welfare criteria (limiting housing deprivation) collected by the Social Development Department of the Gdańsk City Council and the Gdańsk Social Innovation Foundation prove it. In everyday life, participants of the Program develop social competencies, recover from inter and intrapersonal crises, and create positive social relations. According to the literature, there is an increase in QOL also in relation to subjective criteria (Sigry, 2002). Evidence from the social services and aging literature shows that home care delivery is more beneficial for older people and postpones dependency (Alford and O’Flynn, 2012). There is no staff in such apartments to take care of the resident. Instead, some social workers and trainers are responsible for supporting him/her in everyday activities. Such action affects individuals’ mental and physical well-being (Doumit and Nasser, 2010; Ramkissoon, 2020a). Additionally, assisted housing counteracts the harmful effects of the COVID-19 pandemic in terms of global perceived “confinement” in the place of residence. Constant contact of social workers with residents influences the creation of bonds and maintaining social relations, e.g., through regular, meaningful conversations with the elderly (Ramkissoon, 2020a). This was also noticed in multi-person flats for people leaving foster care.

However, the experiences of the homeless do not confirm these findings. They rather concern people in a crisis of loneliness, such as people with disabilities, the long-term sick, or the elderly. Confinement in apartments (as consequence of the spread of the COVID-19 virus) resulted in a decrease in the quality of life in relation to the fulfillment of the need for self-reliance, independence, and self-control among homeless participants of the Program. Being confined during epidemics brought a sense of isolation and emotional distress (Ramkissoon, 2020a). The literature shows that when a significant change of context occurs people may be required to re-invent new ways of performing habitual activities (e.g., Bamberg, 2006; Ramkissoon, 2020a). It is known as “habit discontinuity” that allows changing behavior (Verplanken and Wood, 2006). It is used by people starting their participation in the Program, but it does not apply to people living in the streets or living in housing exclusion. This aspect should be deepened in subsequent studies, but the lack of processual support may be the leading cause of failure.

Participation in GSHP increases professional activity and, above all, meets the basic need for security, related to the constant possession of a flat (Nussbaum and Sen, 1993; Mulder, 2006). Moreover, the data collected by the foundation underlines the value of combining housing support with supported employment. Working in social enterprises run by the foundation generated an increased impact on the inhabitants. Going beyond the usual work of the trainer with the resident, it concerned regular contacts at work, collecting additional information about the psychophysical condition of the program participants and possible progress or difficulties in social and professional reintegration. Social enterprises run a business, at the same time setting social goals and investing the generated surpluses in the community of employees and the local community. Such activities increase the reintegration effectiveness of social projects (Wee-Liang et al., 2005; Bornstein, 2007).

Additionally, the practice of providing social differentiation within settlements is a good move to ensure that the risk of social exclusion for their citizens is reduced. The results of the study indicate that the introduction of a criterion for the management of social housing resources in a commune, concerning the creation of flats and estates scattered throughout the city, is aimed at limiting the emergence of poor or homogeneous districts, e.g., ethnically, racially, threatened with social exclusion and degradation, which additionally increases the quality of life of their inhabitants and prevents exclusion (Halpenny et al., 2002). Older people are usually more motivated to leave their homes and spend their free time outdoors. It is similar to families with small children. The aesthetic appearance of the neighborhood develops emotional ties with the environment (Ramkissoon, 2021). Place affect created in this way improves their general health and well-being (Williams and McIntyre, 2012). Therefore, good environmental conditions have significant benefits in the short and long term. Additionally, researchers argue that spending more time with social interaction leads to more positive emotions, contributing to better welfare outcomes (Lawton et al., 2009; Williams and McIntyre, 2012). Social variation within neighborhoods also fulfills the function of social supervision over seniors or people with disabilities.

The Program also has its limitations. Difficulties in cooperation between residents and trainers or social workers constitute a barrier to the implementation of the goals and tasks of social housing in the commune. Some of the inhabitants of supported flats declare that they do not see the possibility of cooperation with anyone from the Program because the participation results in constant monitoring of their activities, or the progress interferes with their privacy and life. As a result, they lose the possibility to use the apartments. Another barrier is related to the monitoring and evaluation of GSHP. The variety of organizations implementing the Program leads to reporting different and sometimes incomparable data. In the evaluation of the program implementation, no unified tools have been developed so far for measuring the achievements of the organizations implementing. There is also a need for unified tools for assessing the progress of residents (on-going evaluation) and minimal standardization of the applied work methods. A third of the barriers to GSHP operation are the challenges related to achieving the indicator—50 apartments, annually allocated to the Program. It is caused by the difficulties in finding external financing sources for housing investments and the incorporated support programs. The Program, first, needs small apartments because the largest group of participants are people who run one-person households. However, this situation should change as more municipal investments will provide apartments in 2022–2023.

Nevertheless, some limitations of the present study should be considered. Firstly, the presented results concerning the assessment of the quality of life were formulated from the program implementers’ perspective. Their basis was the assessment of its effectiveness and the satisfaction of the residents’ needs. The analyses of the Program participants’ opinions on changes in their quality of life come from the research of the Gdańsk Social Innovation Foundation. This also applies to detailed data on the social and reintegration successes of the inhabitants. Future research should accept the stand of the residents cooperating with other program implementers to a greater extent. Gdańsk Social Innovation Foundation is a large organization that reasonably represents the NGOs operating in the Program. Nevertheless, to obtain full knowledge about the effectiveness of the implemented social support, it is worth measuring the achievements of other organizations performing the task. Additionally, the use of standardized tools for this purpose will provide the possibility of inter-organizational comparisons. The second reservation is related to the above. Quality of life analysis concerned the assessment of objective well-being criteria. It is worth expanding this research with subjective criteria that program participants could provide.

## Conclusion

Gdańsk Social Housing Program combines solutions in the field of housing and social policy on an unprecedented scale. The solution concerning the introduction of the housing with support model is precious. In Poland, “flats with assistance” are a novelty. It is a type of housing where full daily support is not provided. Meetings depend on the needs of residents and concern budget management, becoming independent, help in finding a job, and psychological support in difficult situations. The idea is to support residents in paying their rent regularly and taking care of the apartment’s condition. This solution protects the inhabitants against the loss of flats and housing resources, devastation, and debts (GSHP, 2016). It has the character of social innovation because nowhere have such ideas been designed and implemented in an orderly fashion. This has implications for social theory and practice. The use of this model impacts the development of knowledge in the field of social support (Fernandez-Gavira et al., 2017). It is worth paying attention to the interesting effects of combining the threads of supported employment with social housing (Kemeny, 1995; Borzaga and Defourny, 2001; Scanlon et al., 2015; Tang et al., 2017). Research shows new possibilities to analyze techniques to help and implement social work. It applies both to work in supported flats and flats with assistance. Practical implications relate to the use of the model as a factor of positive effects on increasing housing security in the everyday life of (vulnerable) residents and more effective management of housing resources by municipalities.

However, thoughtful planning of the Program implementation in the coming years will require the development of further strategies. It is about involving new organizations implementing the Program and extending its impact to new categories of people who need housing. A harmonious relationship between residents, places, community organizations, and local government can promote sustainable social development and contribute to sustainable housing development (Ramkissoon, 2020b). It will also build trust and take action to set new strategic goals. People in a housing crisis do not always meet the Program requirements. So it is crucial to consider how to work with the participants of the Housing First project. The challenge is to achieve the long-term behavioral change of homeless people during a pandemic. Here, effective interventions that lead to a change in habitual behavior will be needed (Verplanken and Wood, 2006). There is also a category of people who find it challenging to comply with the rules of participation in the Program. Identifying them as relevant support stakeholders will require additional measures to encourage attempts to achieve housing security. These findings are in line with claims that the influence of a place is important to people’s health and well-being (Ramkissoon, 2020a), which in turn can improve the overall quality of life in local communities (Ramkissoon, 2020b).

## Data Availability Statement

The raw data supporting the conclusions of this article will be made available by the authors, without undue reservation.

## Ethics Statement

Ethical review and approval was not required for the study on human participants in accordance with the local legislation and institutional requirements. The patients/participants provided their written informed consent to participate in this study.

## Author Contributions

The author confirms being the sole contributor of this work and has approved it for publication.

## Funding

This work was supported by the Institute of Sociology of the University of Zielona Góra, Poland.

## Conflict of Interest

The author declares that the research was conducted in the absence of any commercial or financial relationships that could be construed as a potential conflict of interest.

## Publisher’s Note

All claims expressed in this article are solely those of the authors and do not necessarily represent those of their affiliated organizations, or those of the publisher, the editors and the reviewers. Any product that may be evaluated in this article, or claim that may be made by its manufacturer, is not guaranteed or endorsed by the publisher.

## Footnotes

^1^https://baw.bip.gdansk.pl/UrzadMiejskiwGdansku/document/545878/Uchwa/C5/82a-XXXIII_841_21; http://edziennik.gdansk.uw.gov.pl/WDU_G/2017/215/akt.pdf (Accessed July 10, 2021).

^2^https://download.cloudgdansk.pl/gdansk-pl/d/202105170589/zal-nr-2-sprawozdanie-z-realizacji-polityk-programow-i-strategii-wersja-dostepna-cyfrowo.pdf (Accessed June 30, 2021).

^3^http://mopr.gda.pl/projekt/projekt-realizowany-ramach-gdanskiego-programu-mieszkalnictwa-spolecznego/ (Accessed June 18, 2021).

^4^https://bip.brpo.gov.pl/sites/default/files/GPMS%20Forum%2018.10.2019%20w.4_ost%20bez%20film.pdf (Accessed December 3, 2021).
